# Diagnostic Value of Physical Examination, Ultrasound, and Radiography Compared to Computed Tomography in the Evaluation of Nontraumatic Left Lower Quadrant Acute Abdominal Pain

**DOI:** 10.1155/emmi/1681801

**Published:** 2025-06-09

**Authors:** Fakhroddin Kiani, Seyed Mostafa Meshkati Yazd, Fatemeh Zarimeidani, Rahem Rahmati, Nafiseh Shabani Mofrad, Mehdi Vafaei Nia, Reza Shahriarirad

**Affiliations:** ^1^Department of Surgery, Tehran University of Medical Sciences, Tehran, Iran; ^2^Students Research Committee, Shahrekord University of Medical Sciences, Shahrekord, Iran; ^3^Department of Anesthesiology, Shahid Beheshti University of Medical Sciences, Tehran, Iran; ^4^Department of Surgery, Arak University of Medical Sciences, Arak, Iran; ^5^Thoracic and Vascular Surgery Research Center, Shiraz University of Medical Science, Shiraz, Iran; ^6^School of Medicine, Shiraz University of Medical Science, Shiraz, Iran

**Keywords:** acute abdomen, CT scan, emergency medicine, physical examination, radiology, surgery, ultrasound

## Abstract

**Background:** Acute abdominal pain (AAP) is a common complaint of emergency department patients. An accurate diagnosis is even more crucial when AAP is associated with left lower quadrant (LLQ) pain, which has a wide variety of differential diagnoses from self-limiting to life-threatening diseases. This study aimed to evaluate the diagnostic efficacies of physical examination (PE), plain abdominal radiography (PAR), and ultrasonography (US) compared to the computed tomography (CT) scan in patients with nontraumatic LLQ AAP coming into the emergency department.

**Methods:** This prospective cross-sectional study was performed on 220 patients with LLQ-AAP for > 2 h and < 5 days who underwent PAR, US, and CT after PE. An expert surgeon assigned a final diagnosis. Test characteristics, including diagnostic accuracy, sensitivity, specificity, positive predictive value (PPV), and negative PV (NPV), were calculated for PE, PAR, and US, using a CT scan as the gold standard.

**Results:** Among 220 patients (mean age of 48.17; 55.5% female), PE, PAR, and US yielded an overall accuracy of 30.91%, 35.91%, 50.91%, sensitivity of 75.47%, 62.26%, 39.62%, specificity of 16.77%, 27.54%, 54.49%, PPV of 22.35%, 21.43%, 21.65%, and NPV of 68.29%, 69.70%, and 73.98%, respectively.

**Conclusions:** As a result of the highest sensitivity for PE and the highest accuracy for the US, we suggest considering PE as the primary investigation for identifying urgent conditions in patients with LLQ AAP and the US for an accurate diagnosis rather than PAR.

## 1. Introduction

Acute abdominal pain (AAP) could be caused by intra or extra-abdominal conditions and is specified by the location of the pain [1, 2]. AAP is a day-to-day clinical issue in nontraumatic, urgent, and nonurgent patients [3, 4]. Studies have reported that AAP is endured up to 5 days before referring to the emergency department (ED) and is present in 5%–7% of ED patients [2, 4, 5]. Management of this pain typically entails a clinical evaluation through history taking, performing physical examinations (PEs), blood and urine laboratory evaluations, and, if necessary, radiological imaging [6].

Left lower quadrant (LLQ) pain can originate from the digestive system, urogenital system, vascular system, and musculoskeletal system as well [7–9]. Some possible differential diagnoses of LLQ pain include colitis, diverticulitis, inflammatory bowel disease, irritable bowel syndrome, pyelonephritis, renal stone, ectopic pregnancy, ovarian torsion or mass, endometriosis, prostatitis, and also dissection or aneurysm, abscess, hematoma, or hemorrhage, which may be associated with the increase in mortality rate [10–12].

Clinical evaluation, including PE, can distinguish between critical and noncritical patients while having various limitations, such as unusual or unreliable presentations that may be caused by the accessibility of painkillers and reliance on the assessor; as a result, clinical evaluation is often regarded insufficient [4, 13–15]. In addition to these limitations, numerous possible underlying conditions showed the necessity for imaging. However, clinical evaluation plays a crucial role in choosing the optimal primary imaging modality to give an accurate diagnosis while saving time and money [13, 14, 16–18].

A computed tomography (CT) scan with high sensitivity and specificity could early diagnose a wide range of AAP-related diseases and improve patients' management [6, 19]; however, its extensive use has led to attendant radiation dose, costs, and contrast-caused conditions which are discussed among many studies [4, 6, 17, 19, 20].

Plain abdominal radiography (PAR), an inexpensive and widely obtainable diagnostic modality, is usually used as an initial imaging in AAP, which poses low radiation to the patients. Nevertheless, it is ineffective in identifying most disorders due to the low sensitivity [17, 20]. Furthermore, abdominal ultrasound (US), a modality without radiation and with broad availability, can detect diseases more easily than radiography in AAP patients. However, the US is an operator-dependent utility based on expertise. Prioritizing the US before a CT scan is suggested in clinical practice due to its availability and satisfactory positive predictive value. However, a CT scan should still be ordered in negative or undetermined US cases based on the physicians' judgment [4, 17].

Precise and early diagnosis of patients with AAP in critical conditions is necessary for proper and opportune intervention [21–23]. The choice of the most relevant image after complete history-taking, physical assessment, and appropriate laboratory tests in AAP-presenting patients relies on diverse factors that make it challenging for physicians. Several studies suggest healthcare professionals avoid ordering unnecessary imaging procedures, leading to suffering longer from undiagnosed problems besides more exposure to radiation and more economic burden [17, 23, 24]. Because of controversy between each diagnostic pathway, especially imaging, making sometimes wrong diagnoses due to inappropriate orders, and a variety of differential diagnoses of LLQ AAP between self-limiting general pain and life-threatening one [6, 8, 12, 14, 25, 26], we aimed to pioneer to evaluate the diagnostic value of PE, PAR, and US in comparison to the CT scan in patients with LLQ AAP coming to the ED.

## 2. Materials and Methods

This prospective cross-sectional one-year study was conducted among 220 patients with symptoms of LLQ AAP who attended the ED of Valiasr Hospital, Arak, Iran. Inclusion criteria consisted of adult patients (≥ 18 years) presenting with nontraumatic LLQ-AAP persisting for more than two hours and less than five days' duration. Patients dissatisfied with participating in the study and those with simultaneous pain in another area of the abdomen or underlying disease related to the abdominal organs, such as a history of kidney stones, polycystic kidney disease, and horseshoe kidney, were excluded.

A data form was constructed for data collection, consisting of patient data, clinical features, and imaging findings. A provisional diagnosis was made based on the patient's history and clinical evaluation by the treating specialist physicians, consisting of surgical and emergency medicine residents. They prospectively recorded the patient's characteristics, clinical history, and examination findings on a case file. An expert surgeon also separately re-evaluated the patient's PE and history. After clinical assessment at the ED, all consenting patients underwent PAR, US, and CT. Each imaging procedure was independently done and evaluated by a radiologist blinded to the results of other imaging modalities and the patient's examination and findings. The final diagnosis was made by an expert surgeon based on the CT scan as the gold standard. Patients' data were extracted from the forms and medical files and evaluated regarding the diagnostic value of PAR, US, and PE.

Patient information was recorded confidentially, and no cost was imposed on the patient's family. All procedures were done with the patient's prior written informed consent. The study protocol conforms to the ethical guidelines of the 1975 Declaration of Helsinki. The study was reviewed and approved by the ethics committee of the research council of Arak University of Medical Sciences (Ethics code: IR.ARAKMU.REC.1398.133).

Data entry was performed using Microsoft Excel 2019, and all the data were analyzed using Statistical Package for Social Sciences (SPSS) version 22 (Chicago, IL, USA). The Chi-score and *t*-test were used for statistical significance, and the McNemar test statistic was used to compare sensitivity and specificity between single imaging strategies and PE.

## 3. Results

A total of 220 patients with a mean age of 48.17 (SD: 14.67; range: 19–82) and consisting of 122 (55.5%) females were included in our study. Based on CT evaluation, 53 (24.09%) of our patients were positive for nontraumatic LLQ acute abdomen.

Based on the McNemar test, there was a significant association between PE findings and PAR and US findings (*p*=0.006 and 0.001, respectively). Also, there was a significant association between PAR and US findings in diagnosing LLQ nontraumatic AAP (*p*=0.001). The distribution among different evaluation modalities is demonstrated in Table 1, revealing low to moderate agreement between them. Also, Table 2 shows the diagnostic values of PE, PAR, and US in detecting nontraumatic LLQ acute abdomen based on CT-scan as the gold standard.

## 4. Discussion

Adequate and selected tests are crucial to making a precise and timely diagnosis in managing patients with AAP in ED [27]. AAP can demonstrate itself as LLQ-pain with various differential diagnoses and prognoses, making an accurate diagnosis even more critical [10, 11]. Our prospective cross-sectional study aimed to evaluate the diagnostic value of PE, PAR, and US in the management of 220 patients with LLQ AAP. In addition to the meaningful distinct diagnostic value of each method in our study, we found that PE is more sensitive in detecting patients than PAR or US. Still, the US had the highest specificity while demonstrating a high NPV. The most diagnostic accuracy here was contributed to the US as well.

In managing AAP after history-taking, PE is necessary to limit differential diagnoses and provide efficient data to order more tests and plan treatment [3, 28–30]; however, this can be vastly influenced by the examiner's experience [31]. In this study, PE had 75.47% sensitivity, 16.77% specificity, and 30.91% diagnostic accuracy. However, aside from the high achieved sensitivity, because of the low PPV (22.35%) further diagnostic tests and evaluations are needed. Almost the same as our ratio, Gupta et al. [32] found 83.3% sensitivity in PE with 70.9% predictive accuracy for clinical diagnosis, while Gathwal et al. [33] indicated lower sensitivity within greater PPV and diagnostic accuracy in a smaller study. Furthermore, a review study by Gans et al. [4] with 43%–59% and research of Saurav et al. [34] with 71% revealed greater diagnostic accuracy than our assessment, which may have been related to limitations of the examination, ability of physicians, variety of diseases in each study, or the number of cases. Despite the different opinions regarding PE and the low patient and physician confidence in employing and solely basing management on it, still, improving skills on clinical assessment seems vital to distinguish urgent and nonurgent conditions, reveal a possible diagnosis, and select further sensible imaging [4, 13, 31, 35].

PAR has been a widely available, simple, and inexpensive initial diagnostic imaging modality after clinical evaluation for a long time, with lower radiation than CT; however, its value in the evaluation of adult patients presenting to the ED with AAP has been called into question [17, 20, 35]. Our study demonstrated a diagnostic accuracy of 35.91% for PAR, which was lower than the amount of 47%–56% reported by Gans et al. in a review for conventional radiography, including plain chest and abdomen x-rays. According to this review, conventional radiography had a high false-positive and false-negative diagnosis rate [4]. As the diagnostic value of PAR can be greatly influenced by the radiologist's expertise, its sensitivity and specificity in diagnosing AAP range widely among studies from 23.4% to 43% and from 38.4% to 91.1%, respectively [17, 33, 36–38] compared to which our study had a higher sensitivity of 62.26% but a lower specificity of 27.54%. Furthermore, we revealed a significantly lower PPV of 21.43% compared to the conducted studies by Gathwal et al. and Nagurney et al., which reported 86% and 95% PPVs, respectively [33, 37]. On the other hand, our study showed a relatively high amount of NPV near 70%, despite the study done by Nagurney et al. with an NPV of 27% [37]. Owing to test sensitivity, test specificity, and disease prevalence, predictor values can be affected, which may explain these statistical differences. Though, our results emphasize the fact that radiography should be used to rule out and exclude certain diseases rather than to establish a final diagnosis. In a rather large-scale study on 1021 patients, Van Randen et al. mentioned that PAR could be eliminated from the primary diagnostic pathways of patients presenting with AAP because of limitations in adding value and an unaffected level of confidence in the diagnosis [39]. Overall, our study similarly supports the low diagnostic accuracy of PAR, which can be misleading and detrimental due to the cause of delay in therapeutic decision-making.

The US with wide accessibility, no radiation, and no contrast-related disorders is done prior to PAR and CT scan to reach a correct diagnosis [4, 17]. Our US sensitivity of 39.62% and specificity of 54.59% was greatly lower than two large studies by Lameris et al. [22] and Marasco et al. [17] in Europe with a sensitivity range of 61.8%–70% and specificity range of 85%–98.4%, and several smaller studies in Asia [32, 33, 38, 39] with sensitivity from 78.7% to 97.8% which may have contributed to the small number of cases or distribution of AAP-related diagnoses.

The US can be extremely dependent on the operator, limiting its use in clinical practice [40, 41]. Lameris et al. [22] indicated 74% sensitivity for the radiologist-operated US rather than 65%–69% sensitivity for the resident-operated US with and without supervision in a large-scale study. In another large study by Lindelius et al. [42], diagnostic accuracy for surgeon-operated US and nonsurgeon operated US was 64.7% and 56.8%, respectively. Considering operator expertise, the US in this study was done by an expert blinded radiologist who had no information about the patient to prevent provider bias. Moreover, an accurate diagnosis was made in 50.91% of patients by US in our study. Nevertheless, this number was between 72.2% and 90.71% in two other studies [17]. Two different population studies showed that the US could help physicians make the right diagnosis in over 80% of patients [43, 44]. Besides, Siegel et al. [45] found a concordance of US results compared to discharge diagnosis in 80.9% of patients. Concordance in Nural et al. [46] study was 79.3%, and the treatment method had changed in 47% of patients in that study, while it changed in 66% and 41% of patients in two smaller studies that US was one of their parameters [47, 48]. Also, the PPV of US in our assessment was 21.65%, while Gathwal et al.'s study reported that every person with a positive US test was truly diagnosed [33]. Regarding optimum specificity and high NPV of US in our analysis among PE, PAR, and US, as many previous studies [3, 13, 17, 33, 49], our findings suggest using US after clinical evaluation and before CT scan in AAP is an innocuous, economical and time-saving method for the patients, healthcare personnel and system.

The study's limitations include the single-center nature of our research and the possible diagnosis bias, as our gold standard was the CT scan. Further, large-scale and multicentral studies should be performed to evaluate the reliability of each method and its combinational diagnostic accuracy. In the context of personalized medicine, future studies should focus on evaluating potential biomarkers, such as the neutrophil-to-lymphocyte ratio, which is both cost-effective and applicable to various diseases [50, 51]. It is essential to consider different demographic factors of the patients when conducting these studies to enhance the effectiveness of personalized medicine [52].

## 5. Conclusion

In this study, the US had the highest accuracy, specificity, and NPV. In contrast, physical examination demonstrated the highest overall sensitivity and PPV. Therefore, we recommend using PE as the initial investigation for detecting urgent diagnoses in diagnosing patients presenting with LLQ AAP and the US for an accurate diagnosis rather than PAR due to their economical and radiation features compared to CT scans.

## Figures and Tables

**Table 1:** Comparison of diagnostic methods in favor of nontraumatic LLQ acute abdomen.

**Diagnostic method and results**		**PE**		**PAR**		**US**	
		**Positive (*n* = 179)**	**Negative (*n* = 41)**	**Positive (*n* = 154)**	**Negative (*n* = 66)**	**Positive (*n* = 97)**	**Negative (*n* = 123)**

PAR	Positive	129	25	—	—	—	—
	Negative	50	16	—	—	—	—

US	Positive	81	16	52	45	—	—
	Negative	98	25	102	21	—	—

CT scan	Positive	40	13	33	20	21	32
	Negative	139	28	121	46	76	91

*Note:* US, ultrasound; CT, computed tomography Scan.

Abbreviations: PAR, plain abdominal radiography; PE, physical examination.

**Table 2:** Diagnostic values of physical examination, plain radiography, and sonography in detecting nontraumatic LLQ acute abdomen based on computed tomography scan.

Examination		CT scan		Sensitivity	Specificity	Prevalence	PPV	NPV	Accuracy
		Positive; *n* = 53	Negative; *n* = 167						
PE	Positive	40	139	75.47 (61.72–86.24)^∗^	16.77 (11.44–23.31)	24.09 (18.60–30.30)	22.35 (19.57–25.39)	68.29 (54.65–79.38)	30.91 (24.87–37.47)
	Negative	13	28						

PAR	Positive	33	121	62.26 (47.89–75.21)	27.54 (20.93–34.98)	24.09 (18.60–30.30)	21.43 (17.82–25.54)	69.70 (60.07–77.86)	35.91 (29.57–42.63)
	Negative	20	46						

US	Positive	21	76	39.62 (26.45–54.00)	54.49 (46.62–62.20)	24.09 (18.60–30.30)	21.65 (16.01–28.60)	73.98 (68.71–78.64)	50.91 (44.19–57.69)
	Negative	32	91						

*Note:* US, ultrasound; CT, computed tomography scan.

Abbreviations: NPV, negative predictive value; PAR, plain abdominal radiography; PE, physical examination; PPV, positive predictive value.

^∗^The numbers in the parentheses are 95% CIs.

## Data Availability

The data that support the findings of this study are available from the corresponding author upon reasonable request.
